# Systematic Planning of Genome-Scale Experiments in Poorly Studied Species

**DOI:** 10.1371/journal.pcbi.1000698

**Published:** 2010-03-05

**Authors:** Yuanfang Guan, Maitreya Dunham, Amy Caudy, Olga Troyanskaya

**Affiliations:** 1 Lewis-Sigler Institute for Integrative Genomics, Princeton University, Princeton, New Jersey, United States of America; 2 Department of Molecular Biology, Princeton University, Princeton, New Jersey, United States of America; 3 Department of Genome Sciences, University of Washington, Seattle, Washington, United States of America; 4 Department of Computer Science, Princeton University, Princeton, New Jersey, United States of America; Philadelphia, United States of America

## Abstract

Genome-scale datasets have been used extensively in model organisms to screen for specific candidates or to predict functions for uncharacterized genes. However, despite the availability of extensive knowledge in model organisms, the planning of genome-scale experiments in poorly studied species is still based on the intuition of experts or heuristic trials. We propose that computational and systematic approaches can be applied to drive the experiment planning process in poorly studied species based on available data and knowledge in closely related model organisms. In this paper, we suggest a computational strategy for recommending genome-scale experiments based on their capability to interrogate diverse biological processes to enable protein function assignment. To this end, we use the data-rich functional genomics compendium of the model organism to quantify the accuracy of each dataset in predicting each specific biological process and the overlap in such coverage between different datasets. Our approach uses an optimized combination of these quantifications to recommend an ordered list of experiments for accurately annotating most proteins in the poorly studied related organisms to most biological processes, as well as a set of experiments that target each specific biological process. The effectiveness of this experiment- planning system is demonstrated for two related yeast species: the model organism *Saccharomyces cerevisiae* and the comparatively poorly studied *Saccharomyces bayanus*. Our system recommended a set of *S. bayanus* experiments based on an *S. cerevisiae* microarray data compendium. *In silico* evaluations estimate that less than 10% of the experiments could achieve similar functional coverage to the whole microarray compendium. This estimation was confirmed by performing the recommended experiments in *S. bayanus*, therefore significantly reducing the labor devoted to characterize the poorly studied genome. This experiment-planning framework could readily be adapted to the design of other types of large-scale experiments as well as other groups of organisms.

## Author Summary

Microarray expression experiments allow fast functional profiling of an organism's entire genome and significant efforts are devoted to analyzing the resulting data. Available genome sequences are also increasing quickly. However, it is unexplored how to use available functional genomics data to direct large-scale experiments in newly sequenced but poorly studied species. In this paper, we propose a strategy to systematically plan experimental treatments in the poorly studied species based on their model organism relatives. We consider both the accuracy of the datasets in capturing different biological processes and the redundancy between datasets. Quantifying the above information allows us to recommend a list of experimental treatments. We demonstrate the efficacy of this approach by designing, performing and evaluating *S. bayanus* microarray experiments using an available *S. cerevisiae* data repository. We show that this systematic planning process could reduce the labor in doing microarray experiments by 10 fold and achieve similar functional coverage.

## Introduction

To understand the functions of gene products and the interplay between them, significant effort has been spent on performing and analyzing genome-wide expression profiling experiments. Compared to traditional experiments that study protein functions on the single-gene scale, modern high-throughput techniques efficiently characterize expression of the whole genome. One of the most popular techniques is the gene expression microarray, with thousands of expression profiles available for the commonly-studied species. For example, in the Gene Expression Omnibus repository, over 150 datasets comprised of 2400 conditions were available for *Saccharomyces cerevisiae* as of 2007 [1], with data continuing to appear at an enormous rate. These large scale data have been used to accurately predict gene functions [2]–[4], protein-protein physical interactions [5] and functional relationships for yeast [6] and other model organisms [7],[8], as well as human [9]. On the other hand, new genomes are being sequenced at an exponentially growing rate [10], with more than 2,200 genome sequencing projects completed or ongoing to date. These sequencing efforts accelerate our understanding on diverse species, but identifying the gene sequence is not sufficient to define the biological role of its product, and functional annotation of these genomes lags far behind sequencing.

Many of these newly sequenced species are amenable to further experimental study in the lab. The lack of such functional annotation is partly due to the fact that experiments in poorly-studied species are still mainly based on expertise experience or heuristic trials, rather than using a systematic approach based on comparative functional genomics. Although the heuristic approach is useful in directing specific experiments, it is often far from optimal for a systematic functional annotation of all proteins (or at least the majority) in a newly-sequenced genome. Furthermore, experiments that target a specific biological process may also provide accurate functional signal for additional pathways. For example, hyperosmotic shock datasets not only elucidate stress responses, these experiments provide information on regulation of DNA replication initiation because of the cell cycle arrest that occurs under this condition. This functional coverage information is often implicit. We propose here that systematic analysis and quantification of this information in a well-studied species could be the foundation of a systematic experimental design scheme in related poorly-studied species.

In recent years, computationally directed experiments have been applied to different fields. The most prominent application domain is the prediction of protein function with follow-up *in vivo* tests. For example, the prediction results of an ensemble of three algorithms have been used to direct experiments to find genes required for mitochondrial biogenesis [2]. Experiments that detect physical or genetic interactions have also been directed through computationally integrating quantitative genetic interactions and TAP-MS data [11]. The recent development of the Robot Scientist “Adam” marks the state-of-art pipeline of computationally directed studies, which generate hypotheses and experimentally test them [12]. However, these computational efforts have not been extended to direct experiments in a poorly studied species for functional annotation based on existing knowledge in a well characterized species.

In this paper, we developed a systematic approach to recommend experiments for functional annotation of *S. bayanus* (a poorly-studied yeast species) based on the wealth of available gene expression data in the model organism *S. cerevisiae* (baker's yeast). The system identifies experiments that are informative of genes participating in each function and then uses an optimized combination of the predictive power of each experimental treatment with the coverage overlap between treatments to rank a list of experiments that are able to predict the maximum spectrum of biological processes using the minimum number of arrays. Based on functional analysis we estimated that experimentalists can achieve similar functional coverage in the same or related species with less than 10% of the arrays. We further carried out these recommended experiments in *S. bayanus* and the resulting arrays achieved similar functional coverage to all the existing *S. cerevisiae* arrays with a 10 fold reduction in labor. Our approach is readily adaptable to other sequencing-based measurements of expression and to measurements of protein and metabolite levels, and is potentially applicable to other large-scale experiment types.

## Results

**Figure 1:**
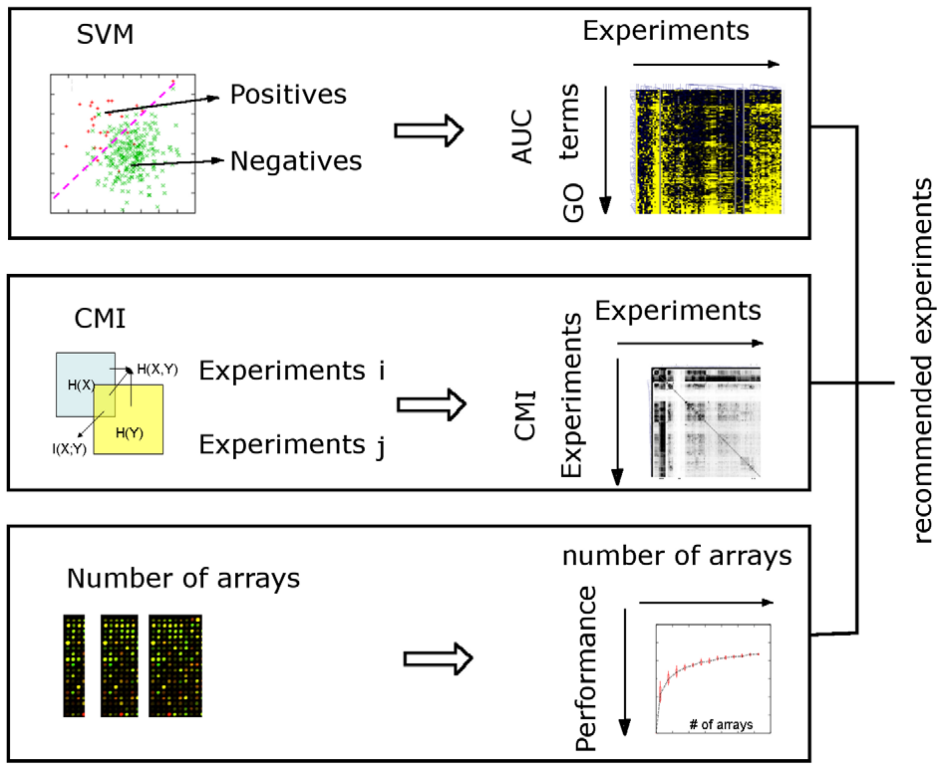
Three-step schematic of the genome-scale experiment planning procedures. First, the informativeness of each experiment in predicting each Gene Ontology (GO) biological process is quantified by bootstrap support vector machine (SVM). Genes in the model organism are grouped into ‘Positives’ (those annotated to the GO under study), and ‘Negatives’ (those not annotated to the GO term). The Area Under the Receiver Operating Characteristic Curve (AUC) of each experiment is estimated by bootstrap SVM, resulting in the GO-experiment matrix. Secondly, conditional mutual information (CMI) was used to quantify the overlap between pair-wise experiments. This results in a symmetric mutual information matrix. Finally, for datasets that contain a large number of arrays, we estimated the minimal number needed to achieve satisfactory function prediction results by a randomized test. The experiment planning system combines the above three aspects and recommends a final list of experimental treatments to be carried out in a related poorly-studied species.

Our experiment planning scheme includes four components (Figure 1): 1. Ranking experiments by their accuracy in predicting a specific biological process; 2. Recommending a list of experiments that maximally covers different functions but shows minimum overlap. 3. Estimating the minimum number of arrays for experiments that consist of a large number of arrays and were originally designed for large-scale characterization of the genome. 4. Finally, combining these three aspects, we recommend a final list of experiments that are optimized for functional coverage of the entire genome; these recommended experiments were carried out and evaluated in *S. bayanus*. Details of these components, as well as computational (through cross-validation) and experimental (through *S. bayanus* experiments) evaluations are presented below.

### Priority of experimental treatments determined by their different coverage of biological processes

Genes play individualized roles in the cell and one gene product can be involved in several different biological processes. Hence for a given experimental treatment or genetic perturbation, we would expect that genes of some functional groups respond more strongly than others. Thus, different datasets are more or less informative of particular processes, including processes that are not necessarily the direct target of the experiment's design. This information, *i.e.*, the informativeness of a dataset when used to predict certain biological process, could be used to select experimental treatments to target certain biological process.

**Figure 2:**
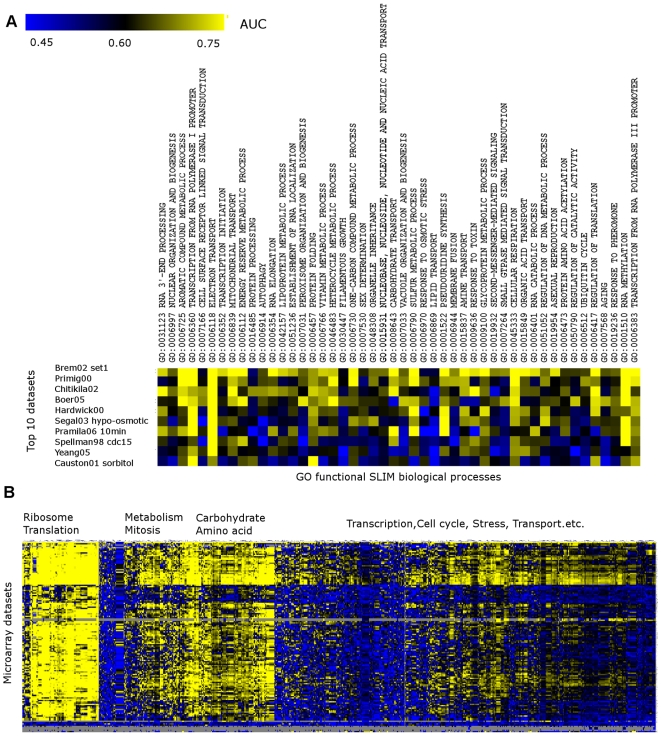
Microarray datasets contain signals for different yet overlapping biological processes. A. The performance (in AUC) of each of the top 10 datasets (in order) recommended by the planning system in predicting different biological processes. B. The performance (in AUC) for the prediction for all GO biological process terms by the entire *S. cerevisiae* microarray repository, clustered by hierarchical clustering. Datasets are very different in their relative performance for different biological processes. Some of the biological processes are well-covered by a variety of experiment treatments, while the majority are only covered by a small fraction of the datasets.

However, this information is often implicit and must be quantified statistically. The Brem *et al.*, 2005 dataset, for example, represents the progeny from an outcross between two strains, executed with the goal of using the resulting expression profiles as phenotypic traits in genetic mapping. It performs well in predicting a wide range of biological processes, including terms not directly related to genetic crossing such as electron transport and sulfur metabolic process (Figure 2A). Our method can rank the candidate experiments according to their informativeness, or how much information each experiment provides on telling whether a gene is related to a certain biological process. We propose to access such informativeness by assessing the predictive performance of a machine learner that uses the data in the experiment under consideration to predict proteins involved in that process. The intuition is that the machine learner will achieve higher accuracy if the training data provides more information about the specific process (at least in terms of functional annotation), and thus this experiment is likely to be highly effective in interrogating this functional group in the evolutionarily related, less well-studied organism. To do this, we use Support Vector Machine (SVM), a state-of-the-art machine learning algorithm [4],[13], though the method can be used with any machine learning approach.

We used bootstrap cross-validation [14] to characterize the performance of our *S. cerevisiae* microarray data collection in predicting Gene Ontology (GO) biological process (BP) terms that are annotated with 10 to 500 genes in *S. cerevisiae* (Figure 2B). We employed several different measurements, including AUC (area under the receiver operating characteristic curve), which characterizes the overall ability of a dataset resulting from an experimental treatment to predict function for proteins in a certain biological process; and precision (accuracy) at 1 percent, 10 percent, 50 percent and 80 percent recall, which focuses more on discovering new genes. It is important to note that these measures assess the ability of experimental treatments to interrogate participants in specific biological processes. Although these measures are influenced by the quality of data as well, we found that they are sensitive to different experimental treatments, because these measures are highly correlated across GO terms in the same treatment between *S. cerevisiae* and *S. bayanus* (see below). If a different property of experimental design is desired, such as ability to assess regulatory interactions or binding partners, this particular characteristic can be optimized through the same evaluation methodology.

With this information, experimental treatments can be ranked by how effective they are in predicting a given biological process. We make this method available to the scientific community through our interactive website, where the users can search for the most relevant experiment(s) for the biological process of interest.

### Redundancy in information provided by different datasets plays an important role in experiment planning

An important phenomenon we observed through the function-dataset informativeness analysis is that some biological processes are well represented in many datasets, while others are only reflected in a small fraction of datasets (Figure 2B). For example, signals for the group of ribosome- and translation-related biological processes are present in the majority of the *S. cerevisiae* expression datasets. Metabolism, mitosis, carbohydrate, and amino acid-related terms are also well represented by many datasets. However, most of the biological processes, for example, transcription, cell cycle, stress and transport-related terms, are only detectable in particular datasets.

Datasets that have the best overall performance in our analysis showed this same range of variability in the terms that they could cover (Figure 2A). For example, transcription from RNA polymerase I promoter (GO:0006360) and electron transport (GO:0006118) are reflected in most of the top 10 datasets. On the other hand, response to toxin (GO:0009636) has strong signal only in the Chitikila02 dataset [15] and the Brem02 set1 data [16], and function of proteins in peroxisome organization and biogenesis (GO:0007031) is well represented only in the Boer05 dataset, which profiles the expression pattern of a leu3 mutant strain [17]. The phenomenon of different functional sensitivity of different expression datasets is consistent with previous studies using different machine leaning methods to estimate informativeness [1],[18]. Therefore, both the accuracy and the redundancy between datasets should be considered for planning experiments.

**Figure 3:**
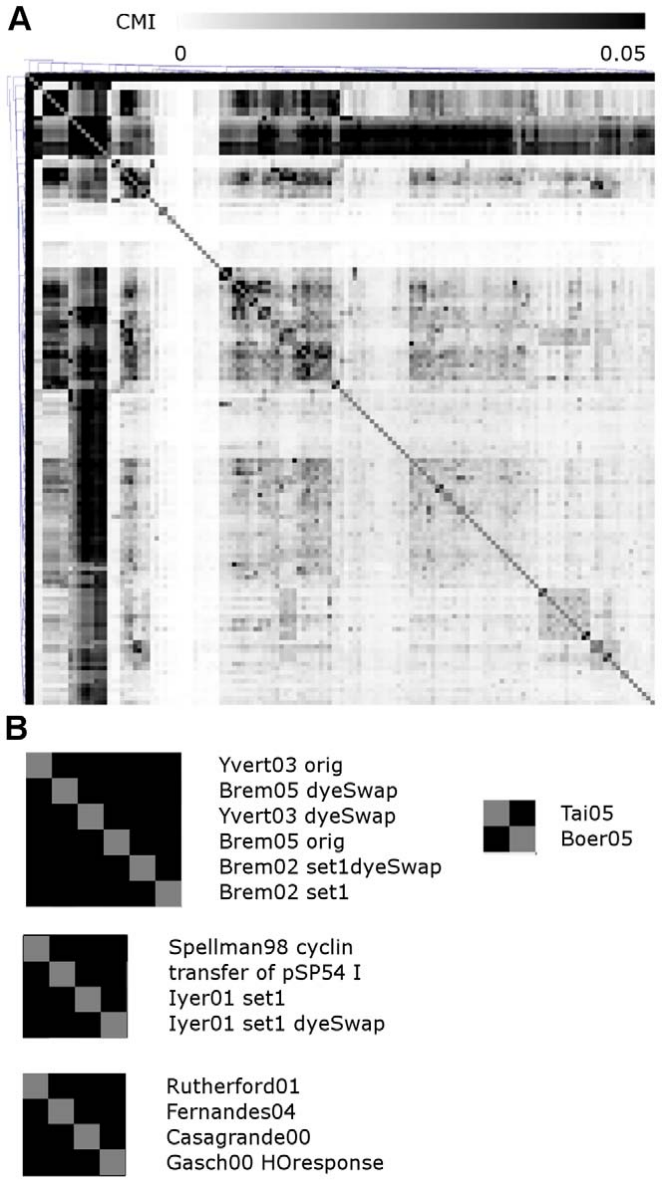
Conditional mutual information could quickly identify redundant datasets in the *S. cerevisiae* microarray repository. A. Overall demonstration of the pair-wise mutual information between datasets, with mutual information values clustered with hierarchical clustering. The mutual information between datasets is highly structured, where black blocks represent several highly overlapping datasets. B. Examples of mutual information between specific datasets. Dataset pairs generated under the same experimental treatment have very high mutual information.

To quantify the overlap in information between datasets, we calculated pair-wise conditional mutual information (CMI). This CMI analysis is highly informative of functional redundancies and therefore is critical to our experiment planning system as shown below. Intuitively, CMI quantifies the overlapping information between datasets in predicting functions. The CMI analysis effectively identified datasets that result from similar experimental treatments (Figure 3). For example, the Brem *et al.* 2002, Brem *et al.*, 2005 and Yvert *et al.*, 2003 datasets have very high mutual information, and in fact are overlapping subsets of the same type of experiment [16],[19],[20]. Less obvious is identification of datasets that are different in their treatments but essentially targeting the same biological processes, for example, the Tai *et al.* 2005 and Boer *et al.*, 2005 datasets have very high mutual information, although the former is a nutrient limitation treatment, while the latter studies the expression in leucine auxotroph mutants. This overlap is likely because both experiments were performed in chemostat culture which may induce similar responses in yeasts. [17],[21]. Less obvious relationships identified by the CMI analysis are those among datasets that do not directly share the same or similar experiment treatments but still contain high mutual information. For example, a set of cell cycle-related experiments are clustered together by their high mutual information, including Spellman98 cyclin [22] and the two technical replicates from Iyer, et al. 2001 [23] (Figure 3). These experiments altered key cell cycle regulators including the cyclins and the transcriptional regulator MBF/SBF. Although these experiments do not analyze a time course of synchronized cells, they measure the transcriptional response to these key regulators and so are very informative about gene expression regulation in the cell cycle. Similarly, stress-response experiments Rutherford01 [24], Fernandes04 [25] and Gasch00 HOresponse [26] are clustered together, despite the fact that they represent diverse experimental treatments, including iron homeostasis, hydrostatic pressure and hydrogen peroxide. The CMI quantification allows us to statistically identify redundant datasets and avoid such redundancies in experimental recommendations for the less-studied species.

### Only a small fraction of some of the large scale datasets is needed for functional annotation

Large-scale microarray experiments designed for characterization of the whole genome tend to be of very high accuracy but often include a large number of arrays. For example, among the top performing datasets in predicting gene functions are the Brem *et al.*, 2005 experiments designed to detect segregation of expression patterns in an outcross [19] and the Hughes *et al.*, 2000 experiments designed for genome-wide mutation analysis [27]. They include 130 and 300 arrays respectively. Although both datasets are among the top recommended experiments, intensive labor is required to repeat these experiments in a new species.

**Figure 4:**
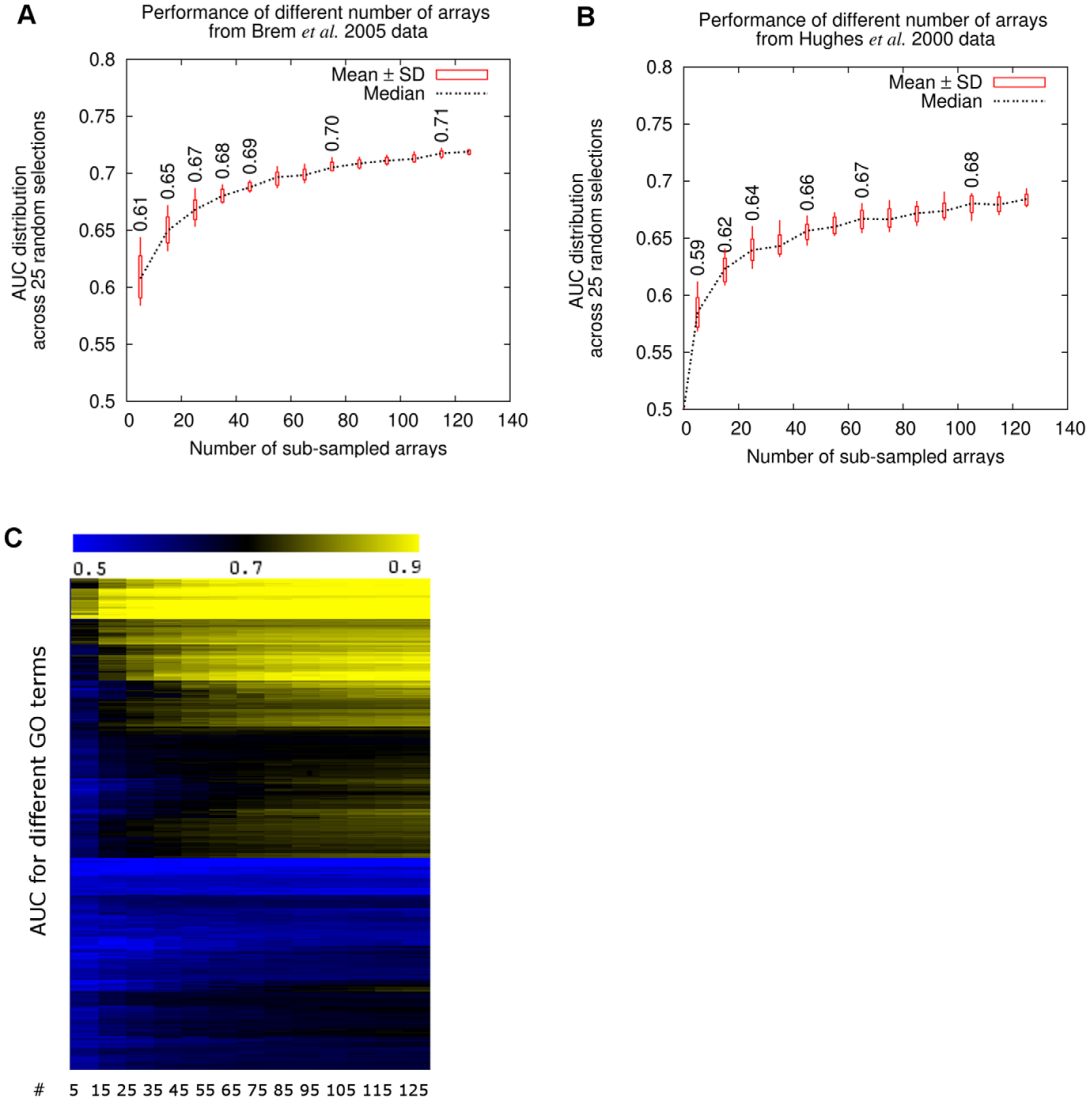
A small number of arrays in some of the very large-scale experiments are sufficient for function prediction. The performance (in AUC) of the random subsets of different numbers of arrays of the (A) Brem *et al.*, 2005 dataset and (B) Hughes *et al.*, 2000 dataset. The mean, median and standard deviation were estimated through 25 sub-samplings. C. The performance (in AUC) of different number of arrays from the Brem *et al.* dataset in predicting different biological processes. The performance of the randomly selected subsets is defined as the average AUC of the GO functional slim biological processes.

We attempted to minimize the number of arrays in these large-scale microarray experiments while retaining their function prediction capability. Through randomized selection of subsets of datasets, we could estimate the accuracy versus number of arrays included in the subsets. Surprisingly, a rather small fraction of the arrays (25–40) can achieve very similar performance in overall function prediction to the entire datasets (Figure 4A and 4B). Additional arrays only add to marginal improvement in performance. Therefore only a small proportion of the arrays of these very large scale experiments are required for our experiment planning system. Of course, this does not mean that these additional data are not biologically relevant, in fact, for genetic linkage experiments the entire dataset is informative. Rather, a subset of these experiments of optimized size can be used for this specific goal of functional annotation; if a different biological question is important, the size of the appropriate subset or entire dataset can be estimated specifically for that question (e.g. regulatory relationship prediction).

Biological processes differ in their sensitivity to the number of arrays required for reasonable assessment of each process (Figure 4C). For example, we could accurately predict the biological process ‘ribosome biogenesis and assembly’ (GO:0007046) with only about 15 arrays from the Brem *et al.*, 2005 [19] dataset. Additional arrays add no improvement in predicting this term. Predictions for many of the other biological processes have different sensitivity to the number of arrays. For example, ‘cellular lipid catabolic process’ (GO:00044242) and ‘histidine metabolic process’ (GO:0006547) could be captured with relatively small number of arrays. On the contrary, ‘co-factor biosynthetic process’ (GO:0051188) and ‘G1 phase of mitotic cell cycle’ (GO:0000080) require a large number of arrays to be well characterized. There are also terms like ‘cell growth’ (GO:0016049), which cannot be captured even using the maximum number of array we tested. For this case, increasing the number of arrays is meaningless. The requirement for the number of arrays on a per-biological process basis was calculated and provided though our online searchable system. For the general recommendation process, we define the minimum number of arrays based on the average AUC across all GO functional SLIM terms [28] and therefore guarantee the overall performance.

### The complete experimental planning scheme effectively captures functions with a limited number of experiments in *S. bayanus*

Our experiment planning system flexibly leverages both the accuracy of each experimental treatment in capturing different functions and the overlap in information between them. We determined the overall accuracy of a dataset by its average AUC across GO functional SLIM terms (as listed in Figure 2B), which are terms curated by biologists to represent functions specific enough for experimental characterization, but which do not have any parent terms satisfying this criterion [28]. The redundancy between datasets was quantified by pair-wise conditional mutual information as described above. A trade-off factor (α) between accuracy and redundancy, where a higher value means more weight on accuracy and vice versa, allows flexibility in the experiment design process. In our study, α was optimized through cross-validation; in the web-interface, the users can optimize this factor according to their specific preferences.

**Figure 5:**
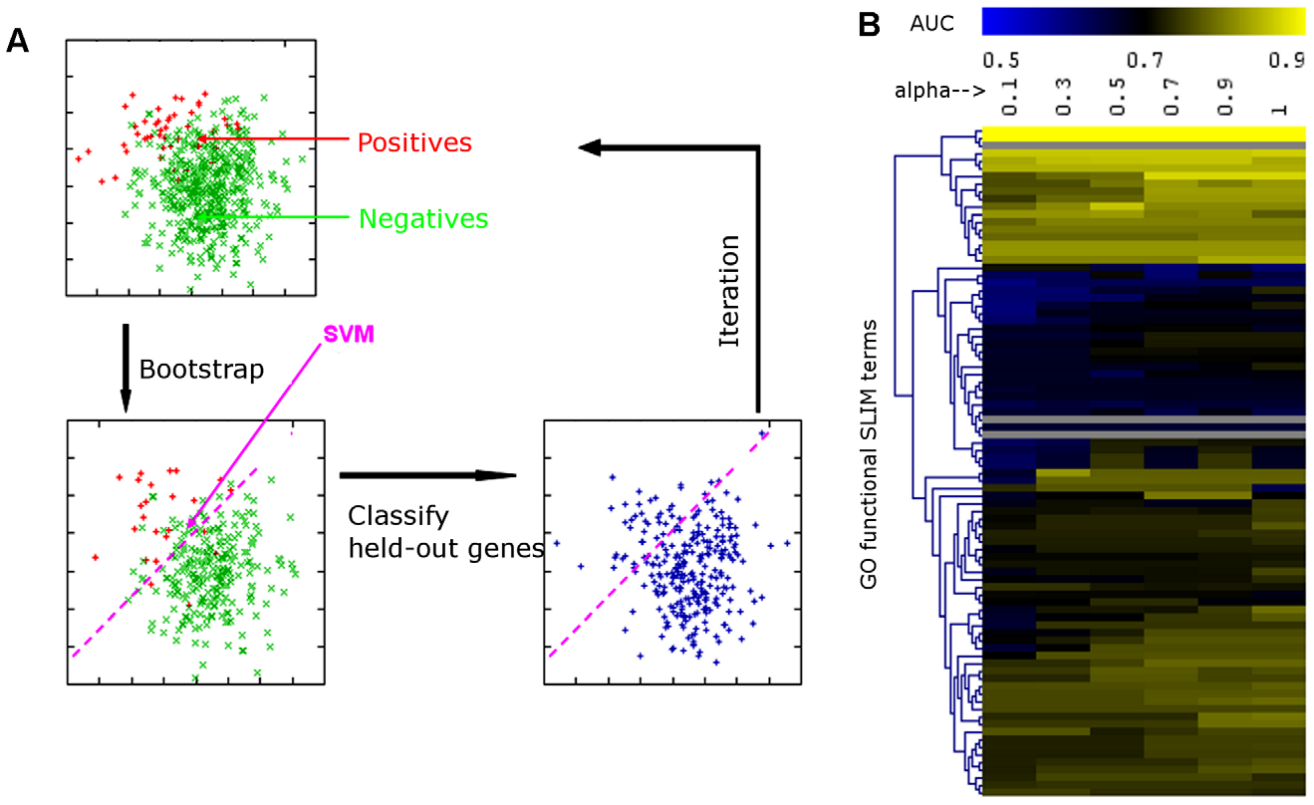
Bootstrap cross-validation determines the trade-off between accuracy and redundancy of datasets. A. A schematic for the bootstrap cross-validation scheme. Using the selected dataset, genes could be placed into hyperdimensional space where support vector machine separates the positive and negative examples (as genes annotated to the GO term and genes not annotated). In each iteration, a set of the genes were bootstrapped as the training set, and the rest remains as the test set. The predicted values of the test set were recorded. After 25 iterations, the median predicted value for a gene when it is in the test sets were taken as the final prediction value for that gene. This value was later used for performance analysis. B. The performance (in AUC) of the top 10 datasets selected by a range of α differs in their ability to predict the GO functional SLIM biological processes. A higher trade-off factor (α) means more weight on the accuracy of the datasets and lower means a heavier penalty is placed on the overlap between them. α = 0.9 achieved the best performance in functional annotation.

Recommendation of datasets for functional annotation requires leverage between data precision and redundancy. We applied bootstrap cross-validation [14] to evaluate the ability of the selected set of data in predicting different functions (Figure 5A). We found that we could optimize the function prediction capability of the top 10 datasets by a trade-off factor α = 0.9 (Figure 5B). Function prediction by SVM maps the original data into feature space, which relies on the accuracy of the datasets and penalized by the redundancy in information between them. Thus an adjustable trade-off factor is necessary and provides flexibility for the experiment recommendation process.

We estimated how effective our approach is in reducing the number of experiments to characterize the overall functionality of the *S. bayanus* proteins based on the *S. cerevisiae* gene expression compendium. We integrated the information from our analysis of the minimum number of arrays in the very-large-scale microarray experiments, and the information from our accuracy and redundancy analysis. This gives us an ordered list of experiments in *S. cerevisiae*, including the number of microarrays that need to be completed in the very large experiments.

We experimentally generated an *S. bayanus* expression data compendium based on the experiments proposed by our system (GEO accession GSE16544). The list of highly informative experiments (250 arrays) included cell cycle progression, meiosis, diauxic shift, nutrient limitation, stress conditions, and outcross progeny.

**Figure 6:**
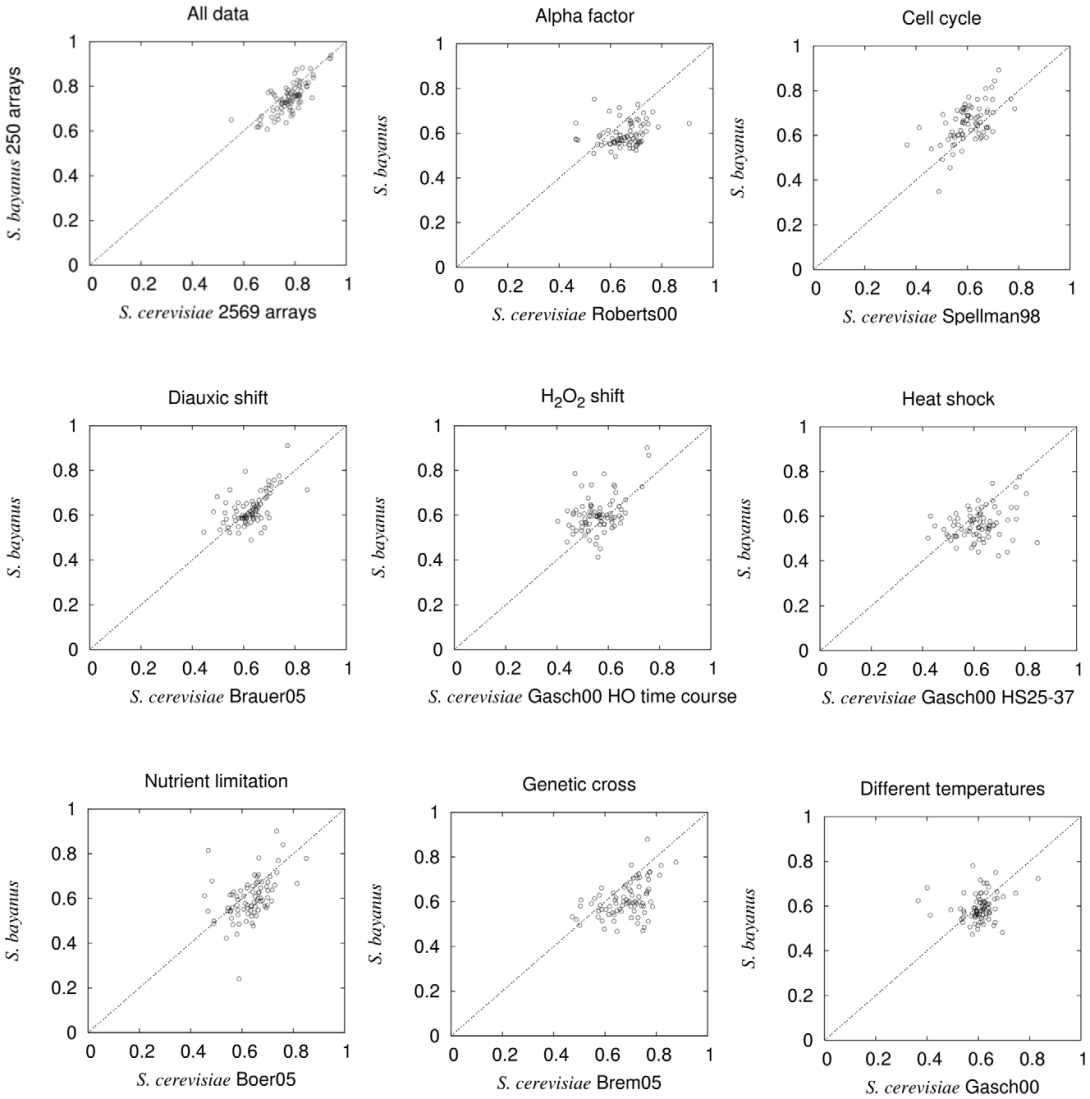
Comparative evaluation of the experimental validation in *S. bayanus*. Each panel depicts the comparison of the performance in AUC between *S. bayanus* and *S. cerevisiae*. GO functional slim terms with more than 30 genes annotated to them were included in all panels. Experimental validation in *S. bayanus* shows that 250 arrays based on the recommendations achieve a similar level of accuracy as 2569 arrays in *S. cerevisiae*. Also shown here are the comparison of performance of eight individually matched experiment pairs in *S. bayanus* and *S. cerevisiae*.

The *S. bayanus* data we generated are highly informative for diverse biological processes (Figure 6). As no *S. bayanus* functional annotation exists, to assess the coverage of *S. bayanus* experimental data, we use the gene ontology annotations from *S. cerevisiae* orthologs of the *S. bayanus* genes. This is a conservative measurement because not all orthologs are conserved in function, but as most genes are likely to be conserved at least on the level of functional annotations, this measure should provide a reasonable lower bound on performance. We used bootstrapping and a linear SVM classifier to estimate the accuracy of the expression data in functional annotation of GO functional SLIM terms [28]. Interestingly, the ‘informativeness’ is highly similar between matched experiments between *S. bayanus* and *S. cerevisiae*, further supporting the validity of our approach (Figure 6).

**Figure 7:**
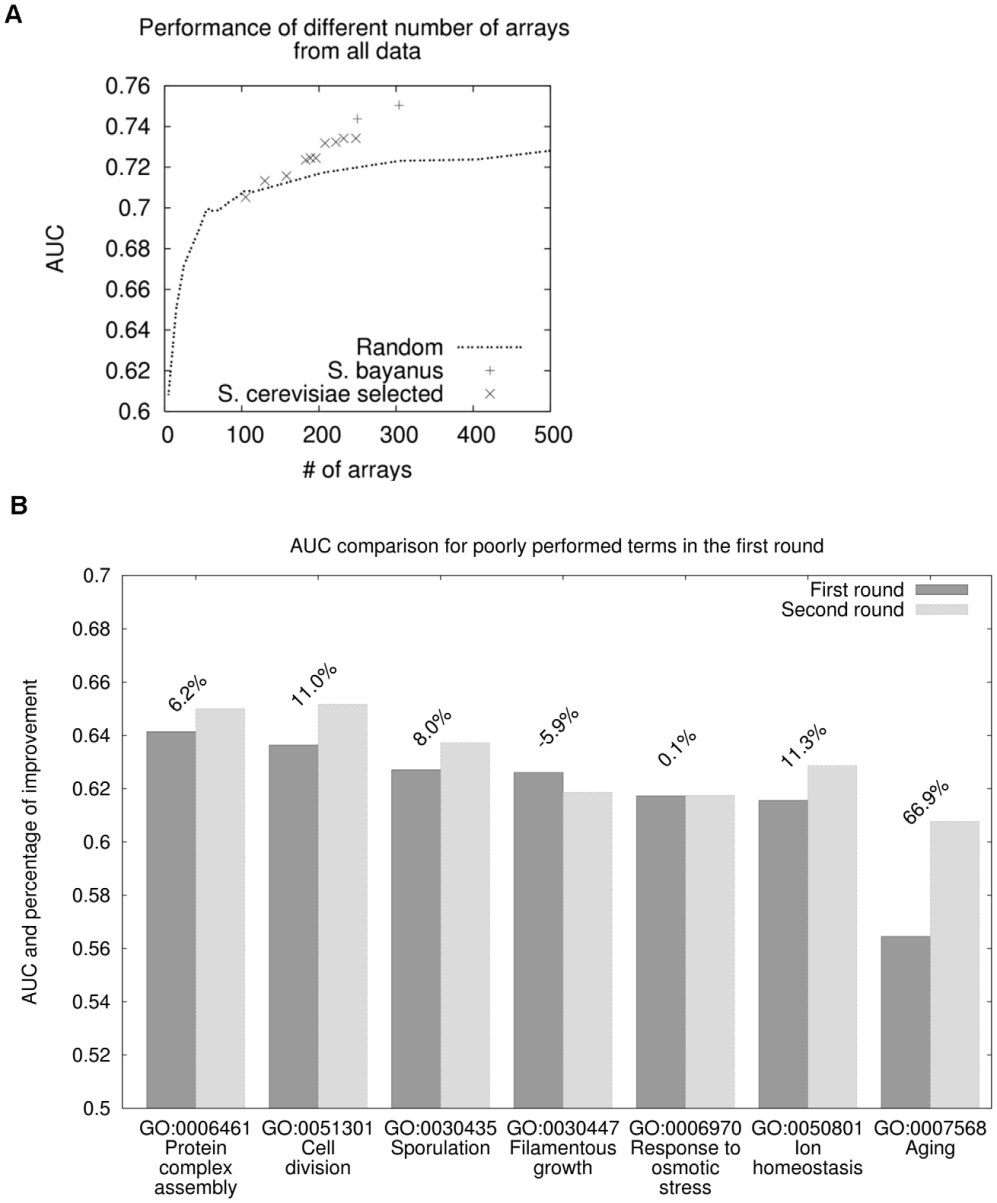
Recommended experiments can more accurately predict functions than a random selection of the data repository. A. Comparison to the performance of randomly selected subsets of the entire expression data repository in *S. cerevisiae*, the recommended datasets, and the recommended experiments carried out in *S. bayanus*. B. Recommended experiments in the second round in *S. bayanus* significantly improved weakly represented terms from the first round. Based on the evaluation results in the first round in *S. bayanus*, we re-designed several microarray experiments for the weakly-predicted terms in the first round. We found that adding these ∼50 experiments to the compendium improved the predictions on the previously weakly predicted terms.

We find that our dataset of 250 *S. bayanus* arrays predicts gene function with an average AUC of 0.74, which is very close to the AUC (0.75) of predictions made with a set of 2547 *S. cerevisiae* arrays (Figure 7A). This is very similar to the theoretical analysis, where we estimated that less than 10% of the arrays of the total 2569 arrays available in the *S. cerevisiae* repository as of 2007 would achieve similar performance. Such performance requires selection of specific experimental treatments – computational simulation shows that selection of random subsets of experiments from the repository substantially decreases overall accuracy (Figure 7). This indicates that the experiment planning scheme can significantly reduce the human and technical resources necessary to characterize a newly sequenced species by providing effective guidance for the most informative sets of experiments for functional annotation based on related model organisms or other well-studied species. To further validate our approach, we also compared the performance of individually matched dataset pairs between *S. bayanus* and *S. cerevisiae* for all GO terms with more than 30 genes annotated to each. The correlation and the similar range of AUC between the two sets (Figure 6) further supports the recommendation of experiments based on closely-related model organisms.

### Evaluation on iterative recommendations in *S. bayanus*

Signals for most of the biological processes are very well represented in our *S. bayanus* expression compendium, to an extent comparable to the theoretical maximum in *S. cerevisiae* (Figure 7A). However, a small set of biological processes, including aging, ion homeostasis, hyperosmotic shock, auxotroph starvation, and alternative carbon sources were not well-captured by the experiments, with an AUC less than 0.65. Thus, we used the system to suggest a second round of experiments targeting these particular processes again based on the accuracy and redundancy analysis.

The second round experiments included 54 arrays covering 11 biological treatments carried out in *S. bayanus* (Text S1). On average we gained a 0.006 (2.5% over random) improvement in AUC over all GO biological process terms with 10 to 300 genes annotated to each. This minor improvement indicates the saturation of the ability to predict functions based on expression data and transfer of annotation through homology. However, we observed an average of 0.012 (10% over random) improvement in AUC in the targeted GO categories, which were poorly predicted in the first round. Five out of seven of the targeted GO categories achieved significantly improved AUC (Figure 7B). Therefore the second round recommends experiments that provide relatively orthogonal information to the first round, indicating the ability of our experimental planning system to extract the information contained in the existing data and to direct further specific experiments.

The top improved terms during the second round (Text S1) are well-explained by the additional datasets. For example, we observed a 52% improvement (0.071) in AUC for “double-strand break repair via nonhomologous end joining” (GO:0006303) and a 56% improvement (0.111) for “DNA catabolic process, endonucleolytic), most likely due to the addition of MMS and zeocin DNA damage datasets. Starvation experiments might explain the 177% improvement in “nitrogen utilization” (GO:0019740) and “histidine biosynthetic process” (GO:0000105). Experiments of alternating carbon sources, particularly glycerol, lead to a 35% improvement in our prediction power on “hexose biosynthetic process.” These observations suggest that our evaluation scheme is well in accordance to the established knowledge in this field.

## Discussion

In this paper, we propose that existing genome-scale data in model organisms could facilitate the planning of experiment treatment in poorly-studied species. We demonstrate the feasibility of this approach by planning microarray experiments for a relatively poorly studied yeast species *S. bayanus* based on an available gene expression data repository for model organism *S. cerevisiae*. In this framework, we recommend an ordered list of experiments targeting the overall functional annotation of *S. bayanus* proteins as well as experiments targeting specific biological processes. We also detected the minimum number of arrays to achieve satisfactory performance for some very large-scale microarray experiments. Our framework results in a substantial reduction in the resources we need to characterize the functions of a poorly-studied genome.

Our work represents the first attempt for large-scale experiment design of a relatively poorly studied organism based on available data in a related organism, rather than existing functional genomics data in that species, which in the case of *S. bayanus* does not exist. This method is complementary to designing experiments based on expert knowledge or intuition, which is irreplaceable when targeting in-depth aspects of biology but is likely not to be optimal for generating a large compendium for functional annotation. Of course, after such initial functional annotation, carefully designed experiments will be necessary to ascertain specific relationships within functions and to further explore the functional space. This task should be facilitated greatly by the availability of the initial functional annotations generated based on the experiment design system and the resulting data compendium.

The experiment design system is adaptable, and can be extended to other related species groups. The current analysis is restricted to *S. cerevisiae* and *S. bayanus*, which are separated from each other by 20 million years. There are thousands of genome projects finished or ongoing, and several model organisms with large amount of genome-scale data. Analysis of these genome-scale data could be used to design experiments for the poorly-studied related species. The 20 millions years distance between *S. cerevisiae* and *S. bayanus* is comparable to the sequence divergence between human and mouse [29]. On the other hand, comparative genomics often focuses on less diverged groups, for example, the *Drosophila* species subgroup or the other *sensu stricto* yeast species. The extendibility to further related groups, however, remains to be validated by future investigation in the intelligent experiment design field. Nevertheless, currently, GO annotations are often transferred between species of vast distance based on sequence alone. Experiment recommendations such as those described here provide a complementary approach to the current annotation scheme. Indeed, expression patterns of the majority of genes are conserved across species over vast distances (e.g. from human to mouse, and from *Candida* to *S. cerevisiae*) [30],[31], suggesting the likely value of applying such experimental design methods across further distances. Applying the experiment design system could not only facilitate the annotation of these genomes but also provide invaluable resources for cross-species expression comparison. In addition, our current study focuses on the prediction of biological processes. The same approach could be extended to molecular function, cellular component and pathway predictions, as well as predictions of particular types of relationships among proteins (e.g. physical, regulatory interactions, etc).

The basic framework could also be extended to other data types, more complex data, and higher organisms. Our current work focuses on microarray data because it is currently the most abundant functional genomics data source for most organisms. This methodology can be readily applied to sequencing-based measurements of expression and to measurements of protein and metabolite level. We expect that as more data of these types become available, applications of this and similar methods will become more common. An extension of this methodology could be developed for other types of large-scale experimental methods, including yeast two hybrid, affinity precipitation, chromatin IP datasets, *etc.* These data, like microarrays, are often readily available in diverse well-characterized model organisms. Furthermore, data from several model organisms could be integrated together so that more confident experiment planning system could be established. Novel methodology that integrates both sequence data and information from these types of large-scale datasets will ultimately allow us to more accurately and quickly understand the differences and similarities of functions between species.

## Materials and Methods

### Microarray data collection and pre-processing

We collected an extensive compendium of *S. cerevisiae* microarray datasets from diverse sources [32]–[36]. This compendium includes 125 datasets with 2569 arrays. A complete list of publications for these datasets is available on the website supporting this publication http://exprecommender.princeton.edu.

To allow reasonable comparison between datasets, we carried out the following normalization steps. For each raw dataset, genes that are represented in less than half of the arrays were removed, and missing values were inserted using KNNimpute [37] with K = 10, Euclidean distance. Technical replicates are averaged, resulting in datasets with each gene followed by a vector representing its expression values in a series of arrays.

### Quantification of dataset-biological process “relatedness” using support vector machines

#### Bootstrap aggregation of SVMs to estimate the accuracy of a dataset

The quantification of the informativeness of datasets to a particular biological process requires a gold standard set of positive and negative examples for this biological process. The positive examples were taken as genes annotated directly to certain biological process or to a descendent of this term. Negative examples were assumed to be all other genes. The basis of our approach is a support vector machine (SVM) classifier. Our previous work has shown that a single linear-kernel SVM often performs better than most of the more complicated machine learning methods in gene function prediction [4]. Therefore, we train a linear-kernel SVM on each biological process.

We applied bootstrap cross-validation which performs better than a variety of cross-validation schemes in error estimation, especially for classes with a limited number of positive examples [14]. Specifically, examples (genes) were randomly sampled with replacement (0.632 bootstrap, meaning the training data will contain approximately 63.2% of the instances) [38]. For each bootstrap sample, a model was learned based on the selected examples, and the resulting classifier was used to give an output on non-selected (out-of-bag) examples. The final classifier outputs were taken as the median of out-of-bag values across 25 independent bootstraps, and the ROC curves were derived from these median values.

We used the SVM^light^ software to implement the SVM classifiers [13]. We have experimented with several parameters and alternative kernels and found that only the cost factor (*j*), by which training errors on positive examples outweight errors on negative examples [39], plays an important role in our function prediction scenario. This is because we are dealing with very unbalanced number of positives (genes annotated to a term) and negatives (genes not annotated to that term). We set *j* as the ratio of negative examples to positive examples as defined above. Therefore, 
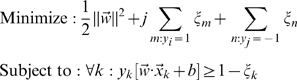

[39] *x_i_* is the feature vector (microarray data values) for gene *i*, *y_i_* equals to 1 or −1 depends on whether gene *i* is annotated to the GO term in study or not. *m* is any cases of the positive examples, and *n* is any cases of the negative examples.

#### Detecting the minimum number of arrays required for very-large-scale experiments

Two of our microarray data in *S. cerevisiae* are of very high accuracy in predicting functions but consist of many arrays (Brem *et al.*, 2005 [19] and Hughes *et al.*, 2000 [27]). To find out the minimum number of arrays required for functional profiling, we randomly selected different numbers of arrays from the original datasets. Specifically, starting from five randomly selected arrays, we progressively increased this number until it reached the total number of arrays in that dataset. For each of the random selections, we used bootstrap cross-validation described above to characterize their performance in predicting individual biological processes. We repeated the random selection for 25 rounds in total, and the average AUC or precision at 1, 10, 20, 50 and 80 percent of recall was used to determine the performance of a specific number of arrays for that dataset.

### Leveraging accuracy and redundancy of data for a final list of experiments

Based on the analysis of experiment performance, we observed that some of the biological processes have strong signal in a wide range of experiments, but others are only sensitive to one or a very limited set of experiments. Furthermore, two very accurate datasets may interrogate a highly overlapping set of processes, thus providing largely redundant information in terms of functional annotation. Therefore, when designing a set of microarray experiments for global function profiling, we should not only consider the accuracy of each experiment in predicting function, but also weigh the overlap in information between datasets. We found using a trade-off factor between the two could allow flexibility in experimental design for different applications (see Results). In the following section, we will describe our measurements of accuracy and redundancy and the combination of the two.

#### Determining the overall accuracy of an experiment

The performance of a specific dataset usually differs when predicting different biological processes. It is therefore necessary to derive an overall measurement of accuracy. Simply taking the average over all GO biological process terms has the disadvantage that not all GO terms are equally informative (some are too general, for example) and the hierarchical structure of gene ontology often makes the performance of one GO term closely related to several others. Therefore, we took the average AUC across all GO functional SLIM terms, which is a set voted by biologists to represent the highest level (in terms of the GO hierarchy) biological process terms that are experimentally relevant for function prediction [28]. This average could reasonably represent the overall accuracy of an experiment.

#### Determining the information redundancy between datasets

Our estimation of the overlap between datasets is based on the conditional mutual information (CMI): 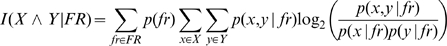
This value is an estimate of the quantity of information shared between dataset *X* and *Y*, given whether genes are functionally related. For a gene pair FR is true (*fr* = 1) when they share at least one co-annotation in the GO functional SLIM terms [28]; it is false (*fr* = 0) when each member of the pair has at least one GO functional SLIM annotation, but they share no co-annotation. In our situation, we calculated the *z*-transformed Pearson correlation *x*, *y* [40] between every gene pair in dataset *X* and *Y* respectively, and mutual information therefore represents the similarity in the distribution of these correlation coefficient values.

Conditional mutual information as calculated above is more suitable than calculating mutual information and ignoring the co-annotation of gene pairs. This is due to the fact that the differences in distribution of correlation coefficient values could be largely due to the presence of more or less ‘functional relationship true/false’ pairs caused by missing genes in the datasets. In CMI, by conditioning on the status of functional relationship between gene pairs, we correct for this bias and only consider the functionally informative part of the mutual information, thus more accurately describing the redundancy between datasets in providing functional information.

### Trade-off between accuracy and mutual information

To leverage the trade-off between accuracy and mutual information, we introduced a trade-off factor α, which linearly combines the two factors: 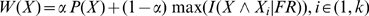
Where *P(X)* is the overall precision of the dataset *X* in consideration, *k* is the number of experiment selected before *X*. This approach allows iterative selection of datasets. Therefore it is suitable for selecting datasets in a species with several experiments available already, which is more likely to be true in the real-world situations. We provide this feature on our website, allowing the user to select the experiments already performed and the desired tradeoff value, so that our system can recommend additional experiments.

### Recommending additional experimental treatment to cover weakly represented biological processes by the first round datasets

We identified GO functional SLIM biological processes that are weakly represented in the first round datasets (below 0.65 in AUC and with a minimum of 30 genes annotated to it in *S. cerevisiae*). This list included 7 biological processes in total (Figure 6). Experimental treatments that best cover each of these biological processes were ranked by accuracy and carried out in *S. bayanus* in the second round.

### Evaluation of the experimental validation in *S. bayanus*

Based on the recommendations, we carried out the experiments in *S. bayanus*. The resulting array data are accessible from the GEO database with accession ID GSE16544. For both first round and second round datasets, we borrowed annotations from *S. cerevisiae* through orthology and applied bootstrap cross-validation to estimate the error rates as describe in *S. cerevisiae* accuracy estimation.

### Web implementation of the experiment design system

We developed a website to facilitate exploration of the functional analysis of the microarray data and our recommendation of datasets for yeast species related to *S. cerevisiae*. This website supports searchable recommendations for datasets targeting specific biological processes and ones targeting the entire functionality of the genome given existing datasets in poorly-studied species. In addition, the number of arrays (on a per biological process basis) required for the large-scale datasets is also searchable by the users. This website is publicly available at http://exprecommender.princeton.edu.

## Supporting Information

Text S1 Supporting information
(0.08 MB DOC)
