# Supplementary material for: Systematic Planning of Genome-Scale Experiments in Poorly Studied Species
Source: PLoS Comput Biol. 2010 Mar 5;6(3):e1000698. doi: 10.1371/journal.pcbi.1000698 (PMC2832676; doi:10.1371/journal.pcbi.1000698)
Supplement: Text S1 — Supporting information (0.08 MB DOC) [file pcbi.1000698.s001.doc]

**Table S1. Second round experiment list.** Based on the computationally identified weakly predicted area based on the first round data, we re-recommended a list of experiments to be carried out in the second round.

| **Treatment** | **Number of arrays** |
| --- | --- |
| Transfer from raffinose to galactose | 5 |
| Transfer from glucose to glycerol | 5 |
| Lysine starvation in auxotroph | 4 |
| MG-132 | 5 |
| MMS | 6 |
| Rapamycin | 6 |
| Hyperosmotic shock with sorbitol | 6 |
| Transfer from glucose to sucrose | 4 |
| Tryptophan starvation in auxotroph | 4 |
| Uracil starvation in auxotroph | 3 |
| Zeocin | 6 |

**Table S2. List of top improved terms in the second round.** Terms with more than 0.05 improvements in AUC are listed below. Terms with most improvement are in accordance with the recommended datasets, further supporting a computationally based approach to select appropriate datasets.

| **GO ID** | **AUC improvement** | **Percentage of improvement over random** | **GO term** |
| --- | --- | --- | --- |
| **GO:0000079** | **0.1560335** | **1.806070799** | **regulation of cyclin-dependent protein kinase activity** |
| **GO:0045859** | **0.1443771** | **1.08801755** | **regulation of protein kinase activity** |
| **GO:0043549** | **0.1443771** | **1.08801755** | **regulation of kinase activity** |
| **GO:0006012** | **0.1124676** | **0.91591662** | **galactose metabolic process** |
| **GO:0000737** | **0.1105309** | **0.562005713** | **DNA catabolic process, endonucleolytic** |
| **GO:0046470** | **0.108791** | **0.483794651** | **phosphatidylcholine metabolic process** |
| **GO:0051338** | **0.1066656** | **0.71625146** | **regulation of transferase activity** |
| **GO:0043085** | **0.1016524** | **2.409921102** | **positive regulation of catalytic activity** |
| **GO:0051049** | **0.0851787** | **0.488228011** | **regulation of transport** |
| **GO:0006488** | **0.0834295** | **0.30854938** | **dolichol-linked oligosaccharide biosynthetic process** |
| **GO:0006817** | **0.0793663** | **0.370522374** | **phosphate transport** |
| **GO:0055065** | **0.0780657** | **0.733954727** | **metal ion homeostasis** |
| **GO:0006875** | **0.0780657** | **0.733954727** | **cellular metal ion homeostasis** |
| **GO:0015698** | **0.0775797** | **0.318416122** | **inorganic anion transport** |
| **GO:0019935** | **0.0758156** | **14.40703861** | **cyclic-nucleotide-mediated signaling** |
| **GO:0019933** | **0.0758156** | **14.40703861** | **cAMP-mediated signaling** |
| **GO:0009065** | **0.0714721** | **0.482086367** | **glutamine family amino acid catabolic process** |
| **GO:0006303** | **0.0714075** | **0.517964367** | **double-strand break repair via nonhomologous end joining** |
| **GO:0006455** | **0.0700161** | **0.231130397** | **translational elongation** |
| **GO:0006442** | **0.0700161** | **0.231130397** | **translational elongation** |
| **GO:0006414** | **0.0700161** | **0.231130397** | **translational elongation** |
| **GO:0031329** | **0.0678803** | **0.389215878** | **regulation of cellular catabolic process** |
| **GO:0006822** | **0.0676923** | **0.252802902** | **anion transport** |
| **GO:0006820** | **0.0676923** | **0.252802902** | **anion transport** |
| **GO:0043162** | **0.0663715** | **0.369268662** | **ubiquitin-dependent protein catabolic process via the multivesicular body pathway** |
| **GO:0019740** | **0.065854** | **1.776802866** | **nitrogen utilization** |
| **GO:0019363** | **0.0654323** | **0.237411001** | **pyridine nucleotide biosynthetic process** |
| **GO:0010038** | **0.0652148** | **0.349560254** | **response to metal ion** |
| **GO:0030004** | **0.0648592** | **0.321547732** | **cellular monovalent inorganic cation homeostasis** |
| **GO:0006790** | **0.0642577** | **0.233336335** | **sulfur metabolic process** |
| **GO:0007231** | **0.0631342** | **0.341258567** | **osmosensory signaling pathway** |
| **GO:0001302** | **0.0611484** | **4.71093443** | **replicative cell aging** |
| **GO:0009076** | **0.0610558** | **0.173442644** | **histidine family amino acid biosynthetic process** |
| **GO:0009075** | **0.0610558** | **0.173442644** | **histidine family amino acid metabolic process** |
| **GO:0006547** | **0.0610558** | **0.173442644** | **histidine metabolic process** |
| **GO:0000105** | **0.0610558** | **0.173442644** | **histidine biosynthetic process** |
| **GO:0046148** | **0.0602279** | **0.211009451** | **pigment biosynthetic process** |
| **GO:0045786** | **0.0600289** | **31.22114734** | **negative regulation of cell cycle** |
| **GO:0051053** | **0.059813** | **0.384353652** | **negative regulation of DNA metabolic process** |
| **GO:0007243** | **0.0584471** | **0.387736825** | **protein kinase cascade** |
| **GO:0030148** | **0.0579163** | **0.19931488** | **sphingolipid biosynthetic process** |
| **GO:0006090** | **0.0567878** | **0.31430477** | **pyruvate metabolic process** |
| **GO:0000920** | **0.0560135** | **0.174920672** | **cell separation during cytokinesis** |
| **GO:0050790** | **0.0545005** | **0.325136198** | **regulation of catalytic activity** |
| **GO:0042274** | **0.0523547** | **0.389547087** | **ribosomal small subunit biogenesis and assembly** |
| **GO:0065009** | **0.052002** | **0.280404219** | **regulation of molecular function** |
| **GO:0046364** | **0.0509608** | **0.358603102** | **monosaccharide biosynthetic process** |
| **GO:0019319** | **0.0509608** | **0.358603102** | **hexose biosynthetic process** |
| **GO:0000165** | **0.050606** | **0.277863647** | **MAPKKK cascade** |
| **GO:0043471** | **0.0505833** | **0.219376565** | **regulation of cellular carbohydrate catabolic process** |
| **GO:0043470** | **0.0505833** | **0.219376565** | **regulation of carbohydrate catabolic process** |
